# Clinical Significance of Early Venous Filling Detected via Preoperative Angiography in Glioblastoma

**DOI:** 10.3390/cancers15153800

**Published:** 2023-07-26

**Authors:** Kotaro Tatebayashi, Noriyuki Nakayama, Daisuke Sakamoto, Tomoko Iida, Shun Ono, Ikuo Matsuda, Yukiko Enomoto, Michihiro Tanaka, Mitsugu Fujita, Seiichi Hirota, Shinichi Yoshimura

**Affiliations:** 1 Department of Neurosurgery, Hyogo Medical University, Nishinomiya 663-8501, Japan; kotarotatebayashi@hotmail.co.jp (K.T.); d.sakamoto.ns@gmail.com (D.S.); matsulica0515@gmail.com (T.I.); poptivo@gmail.com (S.O.); 2 Department of Neurosurgery, Gifu University, Gifu 501-1112, Japan; harukou@gifu-u.ac.jp (N.N.); enomoto@gifu-u.ac.jp (Y.E.); 3 Department of Surgical Pathology, Hyogo Medical University, Nishinomiya 663-8501, Japan; matsudai@hyo-med.ac.jp (I.M.); hiros@hyo-med.ac.jp (S.H.); 4 Department of Neuroendovascular Surgery, Kameda Medical Center, Kamogawa 296-0041, Japan; michihiro.tanaka@gmail.com; 5 Department of Medicine, Graduate School of Medical Sciences, Kindai University, Higashiosaka 577-8502, Japan; mfujita47@gmail.com

**Keywords:** glioblastoma, early venous filling, arteriovenous shunt, vascular mimicry, Avastin

## Abstract

**Simple Summary:**

Our study examined early venous filling (EVF) on angiography, a potential sign of aggressiveness in a type of brain cancer known as glioblastoma (GBM). The study aimed to determine if there were differences in survival rates between GBM patients with and without EVF, and if EVF was associated with a neovascularization process called vascular mimicry. Vascular mimicry occurs when cancer cells form blood vessel-like structures, making the cancer more aggressive and difficult to treat. The study found that GBM patients with EVF generally had shorter survival and were more likely to show signs of vascular mimicry. Simply put, the presence of EVF in GBM patients may indicate a more aggressive form of the disease, which could lead to a worse prognosis. This finding could potentially help physicians predict disease progression and tailor treatment plans. However, more research is needed to confirm these findings and to understand whether they could be used to develop new treatments.

**Abstract:**

Preoperative angiography in glioblastoma (GBM) often shows arteriovenous shunts and early venous filling (EVF). Here, we investigated the clinical implications of EVF in GBM as a prognostic and vascular mimicry biomarker. In this retrospective multicenter study, we consecutively enrolled patients who underwent angiography with a GBM diagnosis between 1 April 2013 and 31 March 2021. The primary and secondary endpoints were the differences in overall survival (OS) and progression-free survival (PFS), respectively, between cases with and without EVF. Of the 133 initially enrolled patients, 91 newly diagnosed with GBM underwent preoperative angiography and became the study population. The 6-year OS and PFS were significantly worse in the EVF than in the non-EVF group. Moreover, 20 GBM cases (10 with EVF and 10 without EVF) were randomly selected and evaluated for histological vascular mimicry. Except for two cases that were difficult to evaluate, the EVF group had a significantly higher frequency of vascular mimicry than the non-EVF group (0/8 vs. 5/10, *p* = 0.04). EVF on preoperative angiography is a robust prognostic biomarker for GBM and may help detect cases with a high frequency of histological vascular mimicry.

## 1. Introduction

Glioblastoma (GBM) consists of heterogeneous glial tumor cells that communicate with each other and the tumor microenvironment [1]. Components of the tumor microenvironment, including tumor cells and tumor-associated stroma, produce various types of molecular mediators that regulate GBM angiogenesis [2]. GBM angiogenesis involves vascular endothelial growth factor (VEGF)-dependent and -independent pathways, including vascular co-option, angiogenesis, vasculogenesis, vascular mimicry, and GBM-endothelial cell transdifferentiation [3]. Vascular mimicry, one of the VEGF-independent pathways, typically occurs in highly invasive, highly metastatic, and advanced malignancies and is often associated with poor patient prognosis [4]. Maniotis et al. reported that vascular mimicry, a tumor microcirculation model found in melanoma over the past 20 years, is a vascular channel-like structure composed of tumor cells but without endothelial cells. It stains positive for periodic acid–Schiff (PAS) and negative for CD31 [5]. In GBM, CD34-negative tumor cells were found within tube-like structures containing red blood cells associated with CD34-positive cells, indicating that the structure was continuous with normal blood vessels [4,6,7]. 

The antiangiogenic agent Avastin (bevacizumab), which targets VEGF, is used in several adjuvant and neoadjuvant cancer therapies [8]. Its efficacy has also been studied in GBM [9]. Despite improvements in progression-free survival (PFS), patients with GBM treated with Avastin eventually develop tumor progression [10,11]. The presence of a VEGF-independent angiogenic pathway is one of the reasons for this. The identification of effective or escape biomarkers may help design trials that combine antiangiogenic agents with agents that target these evasion pathways. 

One of the candidate biomarkers for the evasion pathways is arteriovenous (AV) shunts or early venous filling (EVF). The presence of AV shunts in GBM has been confirmed in a previous study [12]. AV shunts may be a consequence of angiogenesis [13] and can be visualized as EVF by angiography [14]. EVF appears to be present when there is a progression from the arterial filling phase to the venous phase without an intervening arterial emptying or capillary phase. A local or diffuse decrease in circulation time with an orderly progression of the arterial–venous phase also represents an EVF [6]. Only in the latter situation can the term EVF not be used in the same sense as an AV shunt. The presence of an AV shunt and EVF in GBM is thought to (1) increase tumor malignancy by promoting tumor ischemia, (2) increase intraoperative bleeding, and (3) prevent the delivery of chemotherapeutic agents/angiogenesis inhibitors to the appropriate tumor cells and microenvironment [15]. However, the clinical significance and derivation of EVF are not yet fully understood. Therefore, we designed this study to investigate the clinical implications of EVF in GBM as a biomarker for prognosis and vascular mimicry.

## 2. Materials and Methods

### 2.1. Study Design

This retrospective study consecutively enrolled patients with GBM who underwent angiography at Hyogo Medical University and Gifu University. Definitive diagnoses of GBM were made by pathological examination between 1 April 2013 and 31 March 2021. Cases with recurrent tumor, cases with postoperative angiography, and cases with a final diagnosis of non-GBM were excluded. Although MRI is widely accepted as the gold standard for the diagnosis of GBM, our facility utilizes angiography in suspected GBM cases unless patients are ineligible due to systemic or renal limitations or if they initially decline open cranial surgery. This strategy is used to obtain additional information, including confirmation of peripheral vessels, identification of potential arteriovenous malformations (AVMs) and flow-related aneurysms, assessment of the feasibility of preoperative embolization, and/or estimation of potential intraoperative blood loss. Such information plays a key role in formulating safer surgical strategies and reducing operative risks. As angiography is an invasive procedure; we take diligent care to ensure that patients are fully informed and their consent is obtained prior to the procedure. In terms of the treatment strategy, we basically follow the Stupp regimen, which involves surgery followed by postoperative treatment with temozolomide and radiation therapy. Repeated surgery and Avastin are considered as treatment in case of recurrence. The choice of surgical technique was determined by the surgeons at each of the two institutions, although the basic surgical strategy was consistent with the goal of total resection whenever possible. The Institutional Review Boards of both participating centers approved the study protocol, and the requirement to obtain written informed consent from patients was waived owing to the retrospective nature of this study. Instead, a public notice providing information about this study was posted on each center’s website. All patient identifiers have been protected according to ethical guidelines.

### 2.2. Patients and Measurements

**Figure 1:**
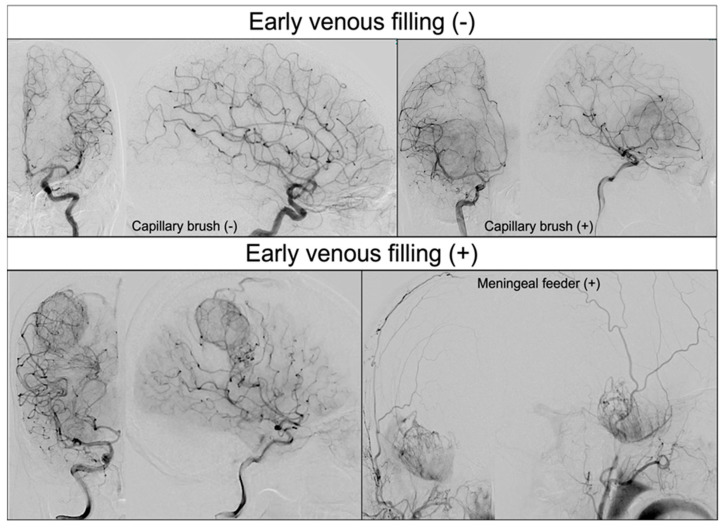
Presence of early venous filling on angiography.

We obtained the following information from each patient’s medical record: age; sex; preoperative Modified Rankin Scale (mRS) score; preoperative Karnofsky Performance Status (KPS); left–right localization of the tumor; brain topography of the tumor; tumor maximum diameter on contrast-enhanced magnetic resonance imaging (MRI); maximum tumor diameter of the high-intensity lesion on MRI fluid-attenuated inversion recovery (MRI FLAIR) images; presence of dural feeder on angiography; presence of EVF on angiography; with or without maximal safe resection; partial or total resection (>90%); adjuvant therapy and its type; final pathological diagnosis; IDH1 mutation by pathology or genetic diagnosis; O6-methylguanine-DNA methyltransferase (MGMT) methylation by pathology or genetic diagnosis; MIB1 index; repeated surgery; and immunohistochemistry for detection of vascular mimicry. Details are described below. The presence of EVF in GBM was confirmed by definitive angiographic visualization of draining veins in the arterial phase (Figure 1). Two independent reviewers (KT and SO) reviewed the angiograms of each patient. Cases in which both reviewers observed EVF were recorded. After an independent evaluation for the presence of EVF, two evaluators discussed cases with conflicting evaluations, and a final evaluation was made. If the patient’s general condition allowed, maximal tumor resection without functional deficits was performed, and the histology of the GBM was confirmed postoperatively. In this study, “pathological diagnosis of MGMT methylation” refers to the use of immunohistochemistry as an alternative technique when polymerase chain reaction (PCR), the gold standard method for detecting MGMT promoter methylation, cannot be performed. A previous report supports the use of immunohistochemistry, stating that it is sensitive and specific enough to detect MGMT promoter methylation status [16].

The primary endpoint was the difference in overall survival (OS) with and without EVF. The secondary endpoint was the difference in PFS with and without EVF. The exploratory endpoint was the relationship between EVF and pathological vascular mimicry.

### 2.3. Histological Evaluation of Vascular Mimicry

**Figure 2:**
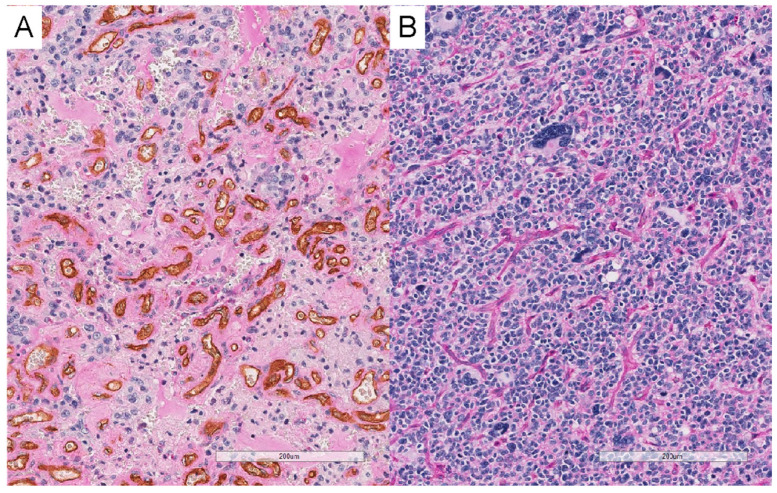
Representative histological images of “brown” and “pink” vessels. Endothelial cells were stained brown by CD34 immunohistochemistry. Bar: 200 μm. (**A**) shows “brown” vessels with stronger CD34 and weaker PAS signals. (**B**) shows “pink” vessels with weaker CD34 and stronger PAS signals. “Pink” vessels were considered to be vascular mimicry.

For the purpose of our histological analysis, we used a software-based (Microsoft Excel version 16.71) random selection method for a total of 20 GBM cases treated at Hyogo Medical University. These cases were equally divided, with 10 demonstrating EVF presence and the other 10 showing no EVF. To minimize bias, the pathologists performing the vascular mimicry evaluation were blinded to EVF status. Visualization of vascular mimicry was achieved by CD34 immunohistochemistry combined with PAS staining, in accordance with methods described in prior literature [7,15]. Two types of small, capillary-like vessels were recognized at low power magnification (×40): “brown” vessels with stronger CD34 and weaker PAS signals (Figure 2A) and “pink” vessels with weaker CD34 and stronger PAS signals (Figure 2B). “Pink” vessels were considered to be vascular mimicry [7,17]. Scoring of “brown” and “pink” vessels was performed for each type of vessel at low power magnification (×40) as follows: score 2 was assigned when the corresponding vessel occupied more than 50% of the tumor area, score 1 when it occupied less than 50% of the tumor, and score 0 when it was not apparent.

### 2.4. Statistical Analysis

Categorical variables are presented as numbers and percentages and were compared using the chi-square test. When the minimum expected count (or frequency) was less than 5, we used Fisher’s exact test. To compare multiple factors of categorical variables, we performed multiple comparisons after factor analysis using the chi-square test. Continuous variables are expressed as the median and interquartile range (IQR); they were compared using the Wilcoxon rank-sum test. The primary and secondary outcomes were compared between the EVF and non-EVF groups. The survival period, defined as the number of months from surgery to death, was censored at the last available follow-up for surviving patients. Kaplan–Meier curves were created to estimate survival in groups classified by the presence of EVF. 

After evaluating the proportional hazard assumption, the differences between the groups were assessed using the log-rank test for 6 years. The effects of the patient group (EVF vs. non-EVF groups) on death were estimated using Cox proportional hazard models and are expressed as hazard ratios (HRs) with 95% confidence intervals (CIs). We adjusted for the following clinically relevant variables to estimate the adjusted HR in the multivariable Cox proportional hazard models: age, baseline KPS, total resection (>90%), and MGMT methylation. To further identify prognostic factors within our study groups, the effects of the patient group (right vs. left side, partial vs. total resection (>90%), and single vs. repeated surgery) on death were also estimated using Cox proportional hazard models and expressed as HRs with 95% CIs, respectively. Using the Cox proportional hazard model, we also performed a subgroup analysis of the adjusted HRs for mortality for patients with and without EVF. The subgroups were as follows: age, baseline KPS, MGMT methylation, and Avastin use. In subgroup analysis, these variables were divided into two categories. 

Because a previous study showed that patients aged ≥ 50 years have a worse prognosis, we dichotomized age with a threshold of 50 [17]. In the preoperative neurological status, the threshold was set at KPS70, not only because it is a neurological threshold of patients’ independence in daily life but also because it is an indication criterion for surgery [18]. Patients with unmethylated MGMT promoters have a poorer prognosis [19]. Previous studies have not shown the efficacy of Avastin on OS [10,11], and to clarify its significance as a biomarker for EVF, we included Avastin use as a factor in subgroup analysis. In our study, all missing values were handled using the listwise deletion method. All statistical analyses were conducted using JMP (version 16.0; SAS Institute Inc., Cary, NC, USA). All reported *p*-values were two-tailed, and statistical significance was set at a *p*-value of < 0.05.

### 2.5. Data Availability

The data supporting the findings of this study are available from the corresponding author upon reasonable request.

## 3. Results

### 3.1. Patient Characteristics 

Of the 133 newly diagnosed GBM patients, 42 (31.6%) who did not undergo preoperative angiography were excluded from the study. Therefore, the analysis was performed on the remaining 91 patients (68.4%) who underwent preoperative angiography (Figure S1). To evaluate the representativeness of our study population, we examined the proportion of patients who underwent preoperative angiography and those who did not, along with their respective backgrounds, prognostic factors, and overall survival (Tables S1 and S2). Patients who underwent preoperative angiography were predominantly male, and a trend toward maximal safe removal was observed in this group. However, no significant differences were observed in terms of preoperative neurological status, postoperative therapy, molecular markers, or overall survival.

The patient characteristics of the study population are shown in Table 1. There were 47 patients with EVF and 44 patients without EVF. The median age of patients with and without EVF at the time of surgery was 66 (IQR, 57–71) and 59 years (IQR, 45–71.8), respectively. There was no significant difference in sex (men: 70.2% vs. 63.6%, *p* = 0.5) or topographical classification (*p* = 0.23) between the groups. However, EVF was observed more frequently when the tumors were located on the right side than when present on the left side (right: 61.7% vs. left: 34.0%, *p* = 0.006). There were no significant differences in the median baseline mRS and KPS between the groups (3 vs. 2, *p* = 0.54, and 70 vs. 70, *p* = 0.39, respectively). The MRI results showed no significant difference in the maximum diameter of the contrast-enhanced images and the maximum diameter of the high-intensity lesions on the FLAIR images between the groups (4.9 cm vs. 4.9 cm, *p* = 0.47, and 7.5 cm vs. 7.8 cm, *p* = 0.74). On angiography, dural feeders were significantly more frequently observed in the EVF group than in the non-EVF group (17.4% vs. 0%, *p* = 0.006). There was no significant difference in the maximum safe removal (93.6% vs. 84.1%, *p* = 0.19), the total (>90%) removal (72.3% vs. 61.4%, *p* = 0.65), adjuvant therapy (*p* = 0.22), and Avastin use (27.7% vs. 29.6%, *p* = 1.0) between the groups. Two patients in the non-EVF group had a poor postoperative course and died within a month, and thus could not receive adjuvant therapy. These two patients were included in the analysis because the original purpose was to provide complete treatment at diagnosis. Regarding the molecular features, we obtained IDH mutation status for 80 of the 91 cases (87.9%). MGMT methylation status was determined for 71 of the 91 cases (78.0%). MIB1 index was obtained in 82 of the 91 cases (90.1%). There was no significant difference in IDH mutation (0% vs. 8.1%, *p* = 0.09), MGMT methylation (26.3% vs. 39.4%, *p* = 0.24), and MIB1 index (30% vs. 30%, *p* = 0.83) between the groups. Whether a pathological or genetic analysis was used to evaluate IDH1 mutations and MGMT methylation is shown in Table S3. The median follow-up duration of the patients with and without EVF was 14 (IQR, 8–21) and 21.5 (IQR, 12–34.8) months, respectively. 

### 3.2. The Outcomes of GBM with or without EVF

**Figure 3:**
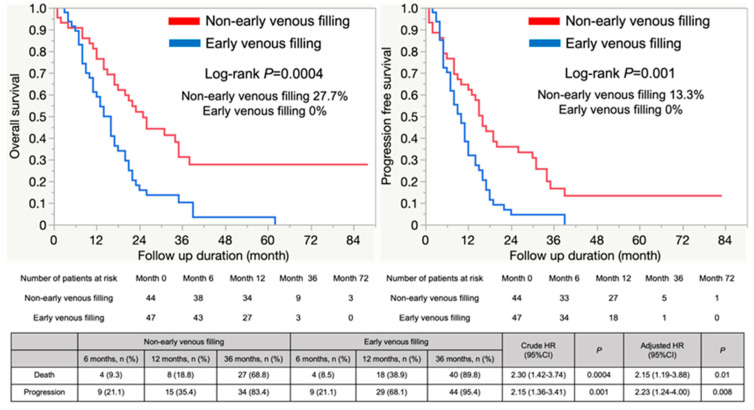
Overall survival and progression-free survival with or without EVF.

Six-year OS and PFS were significantly worse in the EVF group than in the non-EVF group (27.7% vs. 0%, crude HR (cHR): 2.30 (95% CI: 1.42–3.74); adjusted HR (aHR): 2.15 (95% CI: 1.19–3.88); 13.3% vs. 0%, cHR: 2.15 (95% CI: 1.36–3.41); aHR: 2.23 (95% CI: 1.24–4.00), respectively) (Figure 3). 

### 3.3. Prognostic Factors within Our Study Population 

In elucidating the prognostic factors influencing overall survival in our study, a univariate analysis was performed (Table 2). Right tumor localization, total resection (>90%), and the presence or absence of repeated surgery were not identified as prognostic factors (cHR: 1.25 (95% CI: 0.77–2.04), cHR: 0.79 (95% CI: 0.49–1.29), and cHR: 0.98 (95% CI: 0.53–1.78), respectively).

### 3.4. Subgroup Analyses

**Figure 4:**
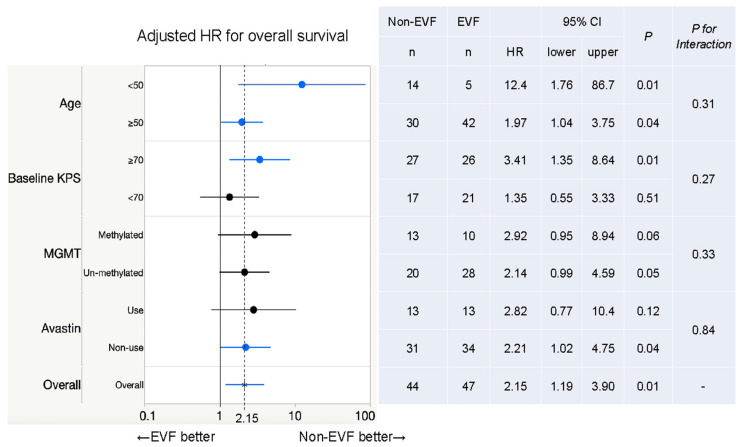
Subgroup analysis. In the forest plot, blue lines represent statistically significant hazard ratios (95% CI does not cross 1) and black lines represent nonsignificant hazard ratios (95% CI crosses 1).

Subgroup analyses of OS at 6 years showed that the EVF group had worse outcomes than the non-EVF group in all subgroups (Figure 4). Among the prognostic factors previously reported and dichotomized variables created in this study, EVF was a particularly clear poor prognostic factor in the subgroups < 50 years and KPS ≥ 70 (aHR: 12.4 (95% CI: 1.76–86.7); aHR: 3.41 (95% CI: 1.35–8.64)). There was no significant difference in prognosis regardless of Avastin use (interaction effect, *p* = 0.84). 

### 3.5. Relationship between EVF and Vascular Mimicry

In a pathological study of the 20 GBM cases treated at Hyogo Medical University, an accurate evaluation was difficult owing to severe necrosis in 2 cases; therefore, the remaining 18 cases were examined. We examined the background factors for the 20 cases that underwent pathological examination (Table S4). No significant differences in background factors were found between the EVF and non-EVF groups. All patients who underwent histological exploration in our study were those who had undergone maximal safe resection. Of the analyzable 18 cases, 10 had EVF and 8 did not. “Pink” vessels, considered vascular mimicry, accounted for more than 50% of the tumor area (score 2) in 5 of the 18 cases. All five patients underwent EVF. The EVF group had a significantly higher frequency of vascular mimicry than the non-EVF group (0/8 vs. 5/10, *p* = 0.04). All scorings of “pink” vessels are shown in Table S5.

## 4. Discussion

### 4.1. EVF and Its Regional Specificity in GBM

Mariani et al. have used high-quality 99mTc-labeled microparticles in patients with glioma. They have demonstrated the existence of an AV shunt in malignant glioma based on the idea that injected microparticles escape capture in the capillaries owing to shunting and are eventually trapped in the lungs [12]. Yoshikawa et al. performed angiography in 26 patients with GBM and used EVF, similar to our study, to detect AV shunts. They reported that 53% of the GBMs had EVF [14]. In our study, 47 of 91 GBMs (51%) showed EVF, which is consistent with the results of previous studies [14]. Given that EVF was observed in approximately half of all GBMs on angiography, we expect it to be a useful threshold preoperative biomarker if its clinical significance is clarified.

The patient backgrounds were similar in the groups with and without EVF, but we found laterality in the presence of EVF. Regarding the laterality in clinical manifestations of GBMs, bleeding is more frequent on the right side than on the left side [20]. Moreover, symptoms are milder on the right side, resulting in delayed diagnosis and reduced quality of life [21]. However, in our study, there was no significant difference in OS between the left and right localizations of GBM (*p* = 0.37). At the molecular level, hemispheric asymmetry was shown in VEGF expression [22]. However, the mechanisms underlying this asymmetry remain unclear. One possible explanation is that GBMs are derived from distinct, region-specific progenitor cells, and genetic differences in progenitor cells determine variations in the biological characteristics of GBMs. Another possibility is that certain genetic alterations may be restricted to specific cells of origin or brain regions [23]. EVF may also have occurred owing to region-specific genetic alterations, which require further investigation.

In our study, 8.9% of all GBMs received blood flow from the dural branches, and EVF was observed in all of these cases. GBM, which is essentially an intra-axial tumor, may affect the microenvironment of marginal angiogenesis beyond the meninges. These results indicate that angiogenesis during GBM is involved in pathological AV shunts and contributes to EVF.

### 4.2. Clinical Significance of EVF

When the GBM progresses beyond 1–2 mm in diameter, the metabolic demands of the tumor cannot be met via diffusion [12]. Hypoxia upregulates proangiogenic factors, such as HIF1α or VEGF, and downregulates antiangiogenic signals. Neoplastic angiogenesis by abnormal blood vessels represented by pathological AV shunts can also lead to perfusion abnormalities [24]. Hypoxia resulting from perfusion abnormalities promotes tumor progression via the activation of angiogenesis, immunosuppression, and metabolic reprogramming [3,25]. Thus, it promotes cell invasion and survival, possibly leading to poor prognosis [25]. EVF is a frequent finding in the angiography of cerebral infarction, which is not the result of a pathological AV shunt but rather of so-called luxury perfusion (a physiological AV shunt), reflecting the marked vasodilation caused by ischemia, regional cerebral hyperemia, and increased regional circulatory velocity [26]. Such pathophysiology may also be involved in GBM as hypoxia and necrosis progress. 

In our study, EVF in GBM was an independent prognostic factor, and subgroup analysis consistently showed that the EVF group had a poorer prognosis in all the subgroups. Additionally, EVF was a clear poor prognostic factor in young patients or patients with good KPS, who are generally considered to have a good prognosis. To our knowledge, the present study is the first report on the relationship between angiographic EVF and prognosis in GBM. As EVF is a poor prognostic factor, we hypothesized that EVF would be a potential therapeutic target. Given the results of our study and the established role of proangiogenic factors such as VEGF in the formation of pathological AV shunts, we then investigated the influence of Avastin, a VEGF inhibitor, on the prognosis of patients with and without EVF. Our goal was to better understand the impact of VEGF-dependent pathways on EVF formation and its potential role as a therapeutic target. However, our findings revealed an unexpected twist in the story.

Our study showed no difference in prognosis regardless of Avastin use between patients with and without EVF. This finding indicates that the presence of EVF cannot be used as a biomarker in the decision to use Avastin. Thus, a VEGF-independent pathway may be involved in EVF formation. We decided to pathologically investigate the existence of a VEGF-independent pathway, vascular mimicry, in the EVF and non-EVF groups.

### 4.3. Relationship between EVF and Vascular Mimicry

In our study, the frequency of vascular mimicry was significantly higher in the EVF group than in the non-EVF group. The cases with a higher frequency of vascular mimicry were present only in the EVF group, but 5/10 (50%) cases in the EVF group were without a higher frequency of vascular mimicry. In other words, although vascular mimicry was not the only cause of EVF, there were no lesions with a high frequency of vascular mimicry in cases without EVF; although both VEGF-independent and -dependent pathways are thought to be involved in EVF, the VEGF-independent pathway is likely to play a partial role in EVF formation. Recently, several studies have focused on identifying strategies that target vascular mimicry-related molecular targets and signaling pathways and inhibit this process [27]. Our results suggest that the presence of EVF on angiography may be useful in determining patient indications for neoadjuvant therapy with a novel therapy targeting vascular mimicry. 

EVF demonstrates the consequences of angiogenic progression in GBM and suggests the malignant potential of the GBM itself. Additionally, EVF may reflect decreased drug delivery and tissue ischemic state and may even indicate vascular mimicry in the tissue.

### 4.4. Potential Efficacy of Preoperative Angiography in GBM Patients

The observed male predominance within our preoperative angiography cohort compared with a non-preoperative angiography cohort may be due to the limitations of our limited sample size. Angiography, used primarily for preoperative planning of craniotomies for tumor removal, is logically consistent with the observed rarity of biopsy procedures. Of note, there was a trend toward a higher rate of total (90%) removal, although not statistically significant, which warrants further investigation. At the very least, angiography does not appear to adversely affect patient outcomes. We believe that angiography provides critical vascular information and has the potential to enhance surgical efficacy and improve patient outcomes. Angiography uniquely facilitates the observation of EVF, and its correlation with vascular mimicry and prognosis on pathologic examination is a novel finding. Our findings highlight the potential of preoperative angiography as a valuable tool in the management of GBM patients, offering diagnostic and therapeutic advantages. Although our study did not specifically analyze MRA data, we consider that future studies examining the relationship between MRA and angiographic and pathologic findings may be beneficial.

### 4.5. Limitations

This study has several limitations. First, as the study design was retrospective, exposure factors and potential confounders may have been poorly controlled. Second, the number of cases was too small to adequately adjust for confounders in the multivariate analysis. Third, the time of angiography, the time of tumor pathology, the time of Avastin use, and the tumor location for pathology sampling were not consistent in all cases. Fourth, the selection of a subset of patients for histological examination was due to resource constraints. The limited number of cases available for the histological study of vascular mimicry might have influenced the robustness of our conclusions. We acknowledge that this induced the potential for selection bias. However, we took care to randomly select patients. When we analyzed the backgrounds of these patients, we found no significant differences between the groups, which further supports our claim of no selection bias in our case selection. However, we understand that despite our efforts, selection bias may exist due to the inherent spatial heterogeneity of EVF. Selective biopsy of sites corresponding to the spatial specificity of angiography may lead to complications such as hemorrhage, and therefore, ethical problems must occur with this method. We believe that a larger multicenter prospective study with consistent temporal and spatial conditions is needed to address these issues. Fifth, this study only focused on vascular mimicry as a VEGF-independent pathway in pathological studies. Therefore, it is difficult to report that the mechanisms of EVF have been adequately discussed. Future studies should focus on other angiogenic mechanisms as causes of EVF development. Sixth, we consider the potential impact of tumor location on prognosis and the need for larger prospective studies to provide more definitive findings on this topic.

## 5. Conclusions

The presence of EVF on preoperative angiography is a robust prognostic biomarker for GBMs. It may also help identify cases with a high frequency of histological vascular mimicry. The results of this study suggest that EVF detection may be useful for patient selection for novel therapeutic interventions for histological vascular mimicry. Further basic and clinical studies are needed to explain angiographic EVF findings from a molecular perspective.

## Figures and Tables

**Table 1:** Patient characteristics.

Patient Characteristics					
		All Glioblastoma (n = 91)	Non-Early Venous Filling (n = 44)	Early Venous Filling (n = 47)	*p*
Age in years, median, IQR		64 (52–71)	59 (45–71.8)	66 (57–71)	0.15
Men, n (%)		61 (67.0)	28 (63.6)	33 (70.2)	0.5
Lesion of tumor					
Right, n (%)		42 (46.2)	13 (29.6)	29 (61.7)	0.007
Left, n (%)		41 (45.1)	25 (56.8)	16 (34.0)	
Middle, n (%)		8 (0.9)	6 (13.6)	2 (4.3)	
Neopallium, n (%)		81 (90.0)	37 (86.1)	44 (93.6)	0.23
Non-neopallium, n (%)		9 (10)	6 (13.9)	3 (4.4)	
Baseline neurological findings					
Modified Rankin Scale, median, IQR		2 (1–4)	2 (1–4)	3 (2–4)	0.54
Karnofsky Performance Status, median, IQR		70 (50–80)	70 (52.5–90)	70 (50–80)	0.39
MRI findings					
Tumor size enhanced lesion, mm median, IQR		4.9 (3.9–5.8)	4.9 (2.8–6)	4.9 (4.1–5.7)	0.47
Tumor size FLAIR high-intensity lesion, mm median, IQR		7.7 (5.6–9.2)	7.8 (5.3–9.3)	7.5 (6.1–9.0)	0.74
Angiographical findings					
Dural feeder, n (%)		8 (8.9)	0 (0)	8 (17.4)	0.006
The degree of removal					
Biopsy, n (%)		10 (11.0)	7 (15.9)	3 (6.4)	0.19
Maximum safe removal, n (%)		81 (89.0)	37 (84.1)	44 (93.6)	
Maximum safe removal	Partial removal, n (%)	20 (22.0)	10 (22.7)	10 (21.3)	0.65
	Total (>90%) removal, n (%)	61 (67.0)	27 (61.4)	34 (72.3)	
Adjuvant therapy					
Non-adjuvant therapy, n (%)		2 (2.2)	2 (4.6)	0 (0)	0.22
Chemotherapy and radiation therapy, n (%)		87 (95.6)	40 (90.9)	47 (100)	
Only radiation therapy, n (%)		1 (1.1)	1 (2.3)	0 (0)	
Others, n (%)		1 (1.1)	1 (2.3)	0 (0)	
Avastin, n (%)		26 (28.6)	13 (29.6)	13 (27.7)	1
Molecular features					
IDH mutation, n (%)		3 (3.8)	3 (8.1)	0 (0)	0.09
MGMT methylation, n (%)		23 (32.4)	13 (39.4)	10 (26.3)	0.24
MIB1 index, median, IQR		0.3 (0.2–0.4)	0.3 (0.2–0.4)	0.3 (0.2–0.4)	0.83
Follow-up					
Reoperation, n (%)		17 (18.7)	9 (17.0)	8 (20.5)	0.67
Follow-up duration, months median, IQR		17 (9–26)	21.5 (12–34.8)	14 (8–21)	0.01

**Table 2:** Prognostic factors within our study population.

	Crude HR (95% CI)	*p*
Right, n (%)	1.25 (0.77–2.04)	0.37
Total (>90%) removal, n (%)	0.79 (0.49–1.29)	0.35
Reoperation, n (%)	0.98 (0.53–1.78)	0.94

## Data Availability

The datasets generated during and/or analyzed during the current study are available from the corresponding author on reasonable request.

## Supplementary Materials

The following supporting information can be downloaded at https://www.mdpi.com/article/10.3390/cancers15153800/s1, Figure S1: Study population; Table S1: Background of groups with and without preoperative angiography; Table S2: Effects of the patient group (preoperative angiography vs. non-preoperative angiography) on death and progression; Table S3: Diagnostic method for IDH mutation and MGMT methylation; Table S4. Background for the 20 cases that underwent pathological examination; Table S5. “Pink” vessels considered to be vascular mimicry.
