# Supplementary material for: Clinical Significance of Early Venous Filling Detected via Preoperative Angiography in Glioblastoma
Source: Cancers (Basel). 2023 Jul 26;15(15):3800. doi: 10.3390/cancers15153800 (PMC10416945; doi:10.3390/cancers15153800)
Supplement: Supplementary file 1 [file cancers-15-03800-s001.zip › cancers-2501933-supplementary.pdf]

Supplementary information

Figure S1. Study population

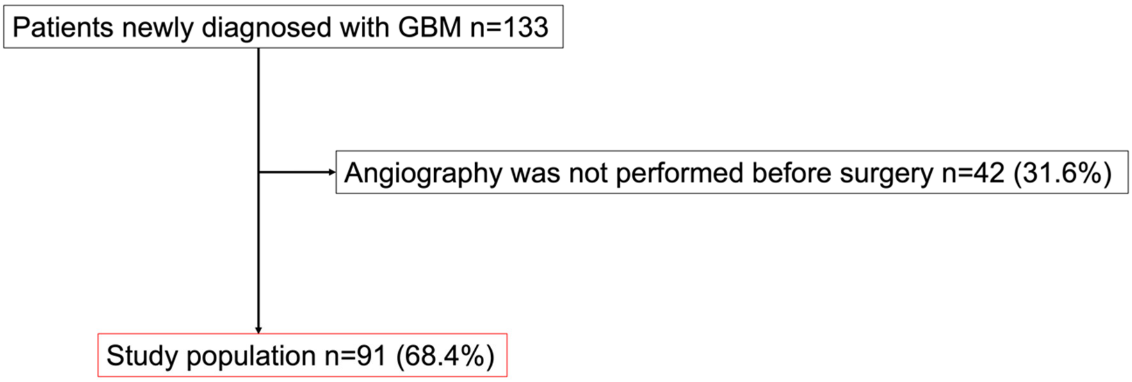

Table S1. Background of groups with and without preoperative angiography

**Patient characteristics**					
		All glioblastoma (n=133)	Preoperative angiography (n=91)	Non-preoperative angiography (n=42)	*P*
Age in years, median, IQR		64 [52–72]	64 [52–71]	67 [52–74]	0.56
Men, n (%)		80 (60.2)	61 (67.0)	19 (45.2)	0.02
**Baseline neurological findings**					
Modified Rankin Scale, median, IQR		2 [2–4]	2 [1–4]	2 [2–4]	0.75
Karnofsky Performance Status, median, IQR		70 [50–85]	70 [50–80]	70 [50–90]	0.85
**The degree of removal**					
Biopsy, n (%)		23 (17.3)	10 (11.0)	13 (31.0)	0.005
Maximum safe removal, n (%)		110 (82.7)	81 (89.0)	29 (69.1)	
Maximum safe removal	Partial removal, n (%)	30 (22.6)	20 (22.0)	10 (23.8)	0.31
	Total (>90%) removal, n (%)	80 (60.2)	61 (67.0)	19 (45.2)	
**Adjuvant therapy**					
Non-adjuvant therapy, n (%)		4 (3.0)	2 (2.2)	2 (4.8)	0.46
Chemotherapy and radiation therapy, n (%)		125 (94.0)	87 (95.6)	38 (90.5)	
Only radiation therapy, n (%)		2 (1.5)	1 (1.1)	1 (2.4)	
Only chemotherapy, n (%)		1 (0.8)	0 (0)	1 (2.4)	
Others, n (%)		1 (0.8)	1 (1.1)	0 (0)	
Avastin, n (%)		44 (33.1)	26 (28.6)	18 (42.9)	0.10
**Molecular features**					
IDH mutation, n (%)		5 (4.2)	3 (3.8)	2 (5.3)	0.70
MGMT methylation, n (%)		32 (31.4)	23 (32.4)	9 (29.0)	0.74
MIB1 index, median, IQR		0.3 [0.2–0.4]	0.3 [0.2–0.4]	0.2 [0.2–0.4]	0.35
**Follow-up duration**					
Follow-up duration, months median, IQR		17 [9–26]	17 [9–26]	14 [9–26]	0.64

Table S2. Effects of the patient group (preoperative angiography vs. non-preoperative angiography) on death and progression

Preoperative angiography vs. non-preoperative angiography	Crude HR (95% CI)	*P*
Death	0.82 (0.55–1.22)	0.33
Progression	0.77 (0.52–1.13)	0.18

Table S3. Diagnostic method for IDH mutation and MGMT methylation

		All (n=91)	Non-early venous filling (n=44)	Early venous filling (n=47)	*P*
IDH mutation	Genetic diagnosis, n (%)	30 (37.5)	13 (35.1)	17 (39.5)	0.69
	Pathological diagnosis, n (%)	50 (62.5)	24 (64.9)	26 (60.5)	
MGMT methylation	Genetic diagnosis, n (%)	30 (42.3)	13 (39.4)	17 (44.7)	0.65
	Pathological diagnosis, n (%)	41 (57.8)	20 (60.6)	21 (55.3)	

Table S4. Background for the 20 cases undergone pathological examination

Patient characteristics			
	Non-Early venous filling (n=10)	Early venous filling (n=10)	*P*
Age in years, median, IQR	45.5 [39.5–70.5]	63 [55.5–70]	0.23
Men, n (%)	6 (60)	5 (50)	0.65
**Baseline neurological findings**			
Modified Rankin Scale, median, IQR	2 [1–2]	3 [1–4]	0.23
Karnofsky Performance Status, median, IQR	70 [70–90]	75 [68–83]	0.46
**The degree of removal**			
Biopsy, n (%)	0 (0)	0 (0)	-
Maximum safe removal, n (%)	10 (100)	10 (100)	
Total (>90%) removal, n (%)	7 (70)	6 (60)	0.64
**Molecular features**			
IDH mutation, n (%)	2 (28.6)	0 (0)	0.07
MGMT methylation, n (%)	3 (50)	2 (20)	0.21
MIB1 index, median, IQR	0.2 [0.2–0.3]	0.3 [0.1–0.4]	0.78

Table S5. "Pink" vessels considered to be vascular mimicry

	All (n=20)	Non-early venous filling (n=10)	Early venous filling (n=10)	*P*
Score 2, n (%)	5 (25)	0	5 (50)	0.02
Score 1, n (%)	7 (35)	3 (30)	4 (40)	
Score 0, n (%)	6 (30)	5 (50)	1 (10)	
Not diagnosable, n (%)	2 (10)	2 (20)	0	
